# Clinical characteristics of snoring patients with primary aldosteronism and obstructive sleep apnea–hypopnea syndrome

**DOI:** 10.1038/s41371-019-0208-9

**Published:** 2019-05-14

**Authors:** Mingyan Li, Qian Ge, Chang-sheng Sheng, Jin Zhang, Hua Li, Wenquan Niu, Xiaofeng Tang, Jianzhong Xu, Ping-jin Gao, Ji-guang Wang, Limin Zhu

**Affiliations:** 1 Department of Hypertension, Ruijin Hospital, Shanghai Jiaotong University School of Medicine, Shanghai, China; 2 International Medical Center of Henan Province, Henan Provincial People’s Hospital, Henan, China; 3 Shanghai Institute of Hypertension, Shanghai Key Laboratory of Hypertension, Ruijin Hospital, Shanghai Jiaotong University School of Medicine, Shanghai, China; 4 Institute of Clinical Medical Sciences, China Japan Friendship Hospital, Beijing, China

**Keywords:** Hypertension, Adrenal gland diseases

## Abstract

The 2016 guideline on the work-up of primary aldosteronism recommended that patients with obstructive sleep apnea–hypopnea syndrome (OSAS) be screened. This study aimed to identify the clinical characteristics of snoring patients with primary aldosteronism (PA) complicated by OSAS. Sixty-eight self-reported or witnessed snoring patients and 609 non-snoring patients diagnosed with PA between 2010 and 2015 were recruited in this retrospective study. Compared to non-snoring patients, snoring patients had significantly (*P* < 0.05) higher body mass index (BMI), diastolic blood pressure (DBP), and serum and urinary sodium, as well as lower estimated glomerular filtration rate (eGFR). Moreover, snoring patients exhibited significantly (*P* < 0.01) higher plasma renin activity levels and lower plasma aldosterone levels and aldosterone-to-renin activity ratios (ARRs) than patients with PA alone. When age, sex, duration of hypertension, and BMI were matched between groups, snoring patients still showed significantly (*P* < 0.05) higher plasma renin activity, serum and urinary sodium, and lower ARR and eGFR than those in the PA-only group. All 68 snoring patients underwent polysomnography, with 7 having mild (apnea–hypopnea index (AHI) ≥ 5 and <15), 21 moderate (AHI ≥ 15 and <30), and 40 severe (AHI ≥ 30) OSAS. The BMI of patients with OSAS was negatively correlated with the lowest SaO_2_ (*r* = −0.318, *P* = 0.018) but not with the AHI. In conclusion, snoring patients with PA tend to have increased BMI and DBP, as well as decreased eGFR and ARR. Snoring patients with PA had higher prevalence of moderate-to-severe OSAS.

## Introduction

Both primary aldosteronism (PA) and obstructive sleep apnea–hypopnea syndrome (OSAS) are common forms of secondary hypertension. Recent studies have found that the prevalence of OSAS among patients diagnosed with PA is 6.7% [1] and reaches 59.4% in PA patients with resistant hypertension [2]. On the contrary, the prevalence of PA among patients diagnosed with OSAS is 34% [3]. Previous studies found aldosterone to be the essential link between PA and OSAS [4]. The severity of obstructive sleep apnea is related to aldosterone status in subjects with resistant hypertension [5]. Under normal physiological status, fluid redistribution occurs during sleep because of the body’s position, resulting in a greater shift to the throat and neck at night. Excessive aldosterone further aggravates submucosal edema in the upper airway during sleep owing to water–sodium retention and elevated blood volume, thereby increasing upper airway resistance and promoting the development of OSAS [6]. Moreover, OSAS induces the activation of the sympathetic nervous system and the renin–angiotensin–aldosterone system (RAAS) by repetitive hypoxemia and hypercapnia at night, leading to worsening blood pressure control and target organ damage in patients with PA [7]. Both PA and OSAS can result in serious cardiovascular and renal diseases, as well as metabolic syndrome. Therefore, the Endocrine Society revised the requirement of PA screening for OSAS patients in the clinical practice guideline for primary aldosteronism in 2016 [8]. To date, there are few studies focusing on the clinical characteristics of patients afflicted with both diseases or on the influence of OSAS on the workup of PA [9]. Loud snoring, nighttime choking or gasping, and daytime excessive sleepiness are the major indicators of OSAS. This retrospective study first aimed to determine the clinical characteristics of PA patients with or without above sleep disorders and to identify the differences in target organ damage, metabolic disorder, levels of renin and aldosterone, and the aldosterone–to–renin ratio (ARR) between both groups, then the profile of patients complicated with OSAS confirmed by the polysomnography (PSG) were analyzed.

## Methods

This retrospective study included hospitalized patients who were diagnosed with PA at the Department of Hypertension, Ruijin Hospital, during 2010–2015.

The diagnostic workup for PA was performed according to the Endocrine Society’s clinical guidelines for PA (2008) [10], as reported in our previous study [11]. Before and during the workup, patients were advised to follow a diet with regular salt intake and to withdraw spironolactone or amiloride administration for at least 6 weeks; non-potassium-sparing diuretics for 4 weeks; and β-blockers, angiotensin-converting enzyme inhibitors, and angiotensin II type 1 receptor blockers for 2 weeks. Non-dihydropyridine calcium blockers and/or α_1_-blockers were prescribed for blood pressure control as necessary. All blood samples were collected at approximately 8:00 a.m. after the patients had slept at the hospital overnight. Plasma and urinary aldosterone were measured simultaneously with serum electrolytes. All measurements were performed in a laboratory accredited by the College of American Pathologists (No. 7217913). Plasma aldosterone concentration (PAC) and plasma renin activity (PRA) were measured using radioimmunoassays following the manufacturer’s instructions (Beckman Coulter). The intra-assay and inter-assay coefficients of variation were 9.3% and 9.5% for aldosterone and 10.1% and 10.2% for renin activity, respectively. The reference values were 29.4–313.3 pg/mL for PAC and 0.1–17.4 ng/mL·h for PRA, respectively. Patients with baseline serum potassium levels <3.5 mmol/L were administered with potassium chloride supplements to ensure that they were normokalemic before testing and adrenal venous sampling.

The diagnosis of PA was based on a plasma aldosterone/renin activity ratio of >240 pg·mL/ng·mL·h from two independent blood samples [12] and confirmed with a saline infusion test in which patients were infused with 500 mL of 0.9% saline hourly for 4 h (between 8:00 a.m. and 12:00 p.m.); PA was confirmed by post-infusion PAC levels >60 pg/mL.

Patients underwent adrenal vein sampling (AVS) if they were candidates for adrenalectomy and if they were willing to receive the operation [10, 13] AVS was conducted without cosyntropin stimulation and with subsequent catheterization. A selectivity index (defined as the concentration ratio of adrenal venous plasma cortisol to peripheral plasma cortisol) ≥3 was considered to indicate correct catheterization. A lateralization index (defined as the ratio of cortisol-corrected aldosterone from the dominant side to that of the non-dominant side) ≥2 was considered to indicate lateralization.

The criteria for unilateral adrenalectomy were mainly based on the 2008 Endocrine Society’s guideline for PA [10] and the 2014 AVS expert consensus [13]. In brief, patients with lateralization index ≥2 after AVS underwent unilateral adrenalectomy. Patients who were aged <40 years, had a florid primary aldosteronism phenotype, and who had clear unilateral adrenal nodular adenoma ≥10 mm and a normal contralateral adrenal gland on computed tomography imaging bypassed AVS and received adrenalectomy directly. No patients were estimated to have a contraindication of surgery.

Patients were interviewed with their sleep status on the day of hospitalization. Patients with self-reported or witnessed loud snoring or apnea during nighttime or daytime sleepiness underwent PSG. They were included in the “PA with snoring” group, other patients were included in the “PA only” group. Patients in these two groups were matched by age, sex, duration of hypertension, and body mass index (BMI) in a ratio of 1:2 for the purpose of further analysis. OSAS was diagnosed based on an apnea–hypopnea index (AHI) of ≥5/h [14].

All patients underwent biochemical analyses of plasma glucose, creatinine, uric acid, electrolytes, renin activity, plasma aldosterone, 24-h urinary aldosterone, and 24-h urine protein, as well as echocardiography. Those with common secondary hypertensive conditions such as renal artery stenosis, renal parenchymal hypertension, Cushing’s syndrome, and pheochromocytoma were excluded. This study was approved by the Ruijin Hospital Ethics Committee, and all participants provided written informed consent.

### Statistical analyses

All data were analyzed by using the SPSS statistical software, version 20.0 (IBM, Armonk, NY). Normally distributed variables were analyzed using Student’s *t* test or analysis of variance test. Variables without Gaussian distributions were analyzed using Student’s *t* test if the data met Gaussian distribution after logarithmic transformation; otherwise, a non-parametric test was used. The *χ*^2^ test was used for nominal data. Pearson correlation analysis was used to determine the relationship among parameters. All normally distributed variables are presented as means ± standard deviations, and variables that did not exhibit a Gaussian distribution are presented as medians. Statistical significance was considered at *P* < 0.05.

## Results

### Comparison between patients with PA and snoring vs. those with PA only

A total of 677 patients with PA were recruited, including 68 who had self-reported or witnessed loud snoring, apnea, or daytime sleepiness. Compared to the control group, patients with PA and snoring had higher proportions of men; obese patients/higher BMIs; those taking higher numbers of antihypertensive medications; higher diastolic blood pressure, triglyceride levels, low-density lipoprotein, creatinine, serum sodium, and 24-h urinary sodium; and lower estimated glomerular filtration rate (eGFR) levels (Table 1). Overall, 25 out of 187 (13.4%) resistant hypertension patients with PA had snoring. There was no difference in the prevalence of resistant hypertension between the groups.

**Table 1:** Comparison of baseline characteristics between the PA and PA with snoring group

	PA (*n* = 609)	PA with snoring (*n* = 68)	*P* value
Age (years)	49.7 ± 11.5	48.9 ± 0.2	0.364
Male sex (%)	337 (55.4)	48 (70.6)	0.017
BMI (kg/m^2^)	24.7 ± 3.3	27.5 ± 3.7	<0.001
Hypertension duration (years)	10 (4–15)	10.0 (5.0–16.5)	0.388
Antihypertensive drugs (*n*)	3 (2–3)	3 (2–4)	0.034
Resistant hypertension (%)	162 (26.6)	25 (36.8)	0.075
24 h SBP (mm Hg)	136 ± 14	137 ± 13	0.440
24 h DBP (mm Hg)	86 ± 9	89 ± 9	0.006
Daytime SBP (mm Hg)	139 ± 14	140 ± 14	0.521
Daytime DBP (mm Hg)	88 ± 9	91 ± 9	0.023
Nighttime SBP (mm Hg)	129 ± 17	132 ± 15	0.275
Nighttime DBP (mm Hg)	81.35 ± 10.44	85.59 ± 9.79	0.002
Serum K^+^ (mmol/L)	3.2 (3.0–3.55)	3.3 (3.0–3.5)	0.984
Serum Na^+^ (mmol/L)	141 (139–143)	142 (141–144)	<0.001
24 h urinary Na^+^ (µg/24 h)	157 ± 71	194 ± 88	0.002
Fasting glucose (mmol/L)	5.20 (4.80–5.70)	5.20 (4.84–5.71)	0.632
2 h postprandial glucose (mmol/L)	7.92 ± 3.25	8.42 ± 3.14	0.065
HbA_1_C (%)	5.60 (5.20–6.20)	5.60 (5.28–6.00)	0.552
Triglyceride (mmol/L)	1.65 ± 1.06	2.01 ± 1.53	0.007
LDL-C (mmol/L)	2.67 ± 0.75	2.90 ± 0.70	0.014
Plasma creatinine (mmol/L)	70.36 ± 19.55	77.76 ± 19.84	0.002
eGFR (mL/min·1.73 m^2^)	101.71 (86.29–116.11)	93.27 (82.07–109.28)	0.017
24 h urinary protein (mg/24 h)	111 (89–152)	120 (96–184)	0.084
LVMI (g/m^2^)	114.33 ± 27.40	116.86 ± 23.36	0.319

Values are indicated as means ± standard deviations or as medians (25th, 75th)

*PA* primary aldosteronism, *BMI* body mass index, *SBP* systolic blood pressure, *DBP* diastolic blood pressure, *HbA*_*1*_*C* glycated hemoglobin A_1_C, *LDL-C* low-density lipoprotein, *eGFR* estimated glomerular filtration rate (Modification of Diet in Renal Disease [24]), *LVMI* left ventricular mass index [25]

As shown in Table 2, PRA at the supine and standing positions in patients with PA with snoring was significantly higher than in patients with PA alone (*P* < 0.001); the proportion of renin activity >1 ng/mL·h in the standing position was also higher in the former group than the latter. The plasma aldosterone in the standing position and 24-h urinary aldosterone levels were relatively lower in the PA with snoring group; and there were significant differences in the plasma aldosterone levels at the supine position and in the ARR value at the standing and supine positions between groups (*P* < 0.01).

**Table 2:** Comparison of renin, aldosterone, and ARR between the PA group and PA with snoring group

	PA (*n* = 609)	PA with snoring (*n* = 68)	*P* value
Supine renin activity (ng/mL·h)	0.30 ± 0.37	0.70 ± 0.74	<0.001
Standing renin activity (ng/mL·h)	0.85 ± 1.05	1.45 ± 1.31	<0.001
Supine plasma aldosterone (pg/mL)	325 ± 243	247.49 ± 136.26	0.009
Standing plasma aldosterone (pg/mL)	316 ± 2067	282.25 ± 181.44	0.128
Standing renin activity >1 ng/mL·h (%)	27.6	52.9	<0.001
Supine ARR ([pg/mL]/[ng/mL·h])	1371 (619–3933)	481 (257–564)	<0.001
Standing ARR ([pg/mL]/[ng/mL·h])	1678 ± 5376	490 ± 806	<0.001
24 h urinary aldosterone (µg/24 h)	22.4 ± 16.3	19.3 ± 9.9	0.275

Values are indicated as means ± standard deviations or as medians (25th, 75th) or as percentage

*PA* primary aldosteronism, *ARR* plasma aldosterone-to-renin activity ratio

In total, 260 (42.7%) patients with PA alone and 31 (45.6%) patients with PA and snoring received AVS (*P* > 0.05). Respectively, 159 (61.2%) and 18 (58.1%) patients showed lateralization (*P* > 0.05). Of the 244 patients with PA alone who received unilateral adrenalectomy (surgery rate: 40%), pathological subtyping revealed that 126 (51.64%) had adenoma, 116 (47.54%) had hyperplasia, and 2 (0.82%) had an unknown histology. In patients with PA and snoring, 15 underwent surgery (surgery rate: 22%): 1 (6.67%) had adenoma, and the remaining 14 (93.33%) had hyperplasia. The differences in operation rate and pathological subtypes between the 2 groups were significant (*P* < 0.01).

### Comparison of patients with PA and snoring vs. those with PA only after matching of characteristics

After matching according to age, sex, duration of hypertension, and BMI, 121 patients with PA only and 66 with PA and snoring patients were analyzed (2 patients with PA and snoring could not be matched owing to excessive BMI and were thus excluded). No significant differences in blood pressure, blood glucose level, triglycerides, low-density lipoprotein, or 24-h urinary protein levels were observed between these 2 subgroups. Nevertheless, the PA with snoring subgroup showed significantly higher serum sodium and 24-h urinary sodium levels, as well as lower eGFR, than the PA-only subgroup. There was no significant difference in plasma and urinary aldosterone levels between the two groups; however, compared to PA-only patients, those with PA and snoring showed significantly higher renin activity levels, higher proportions of renin activity >1 ng/mL·h at the standing and supine positions, and lower ARRs (*P* *<* 0.05; Table 3). Furthermore, 40 patients of the PA-only subgroup and 15 patients of the PA with snoring subgroup underwent unilateral adrenalectomy. There was a significant difference in pathological subtypes between these 2 subgroups, accounting for adenoma (42.5% vs. 6.67%) and hyperplasia (57.5% vs. 93.33%) (*P* < 0.05).

**Table 3:** Comparison of renin, aldosterone, and ARR between the PA and PA with snoring group matched for age, sex, duration of hypertension, and body mass index

	PA (*n* = 121)	PA with snoring (*n* = 66)	*P* value
Serum Na^+^ (mmol/L)	141.8 (139,143)	143 (141,144)	<0.01
24 h urinary Na^+^ (µg/24 h)	168.83 ± 75.68	195.52 ± 88.02	0.035
Supine aldosterone (pg/mL)	249 (166–372)	215 (156–300)	0.090
Standing aldosterone (pg/mL)	265 (191–367)	230 (165–344)	0.116
Supine renin activity (ng/mL·h)	0.20(0.1–0.4)	0.36 (0.16–0.99)	0.001
Standing renin activity (ng/mL·h)	0.58 (0.31–1.13)	1.06 (0.52–2.0)	0.001
Standing renin activity >1 ng/mL·h (%)	27.4	51.5	0.001
Supine ARR ([pg/mL]/[ng/mL·h])	1090 (485–3218)	511 (258–1592)	<0.001
Standing ARR ([pg/mL]/[ng/mL·h])	466 (185–1006)	287 (131–478)	0.001
24 h urinary aldosterone (µg/24 h)	18.9 (13.0–26.5)	16.39 (11.2–26.7)	0.315

Values are indicated as medians (25th, 75th) or as percentage

*PA* primary aldosteronism, *ARR* aldosterone-to-renin activity ratio

### Clinical characteristics of patients with PA and OSAS

**Fig. 1:**
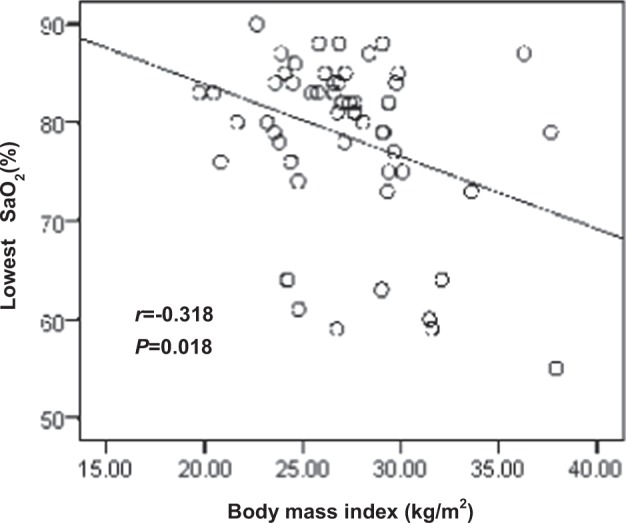
Correlation analysis between body mass index and the lowest oxygen saturation (SaO_2_) level

The 68 patients with PA and snoring underwent PSG. Seven (10%) had mild OSA, 21 (31%) had moderate OSA, and 40 (59%) had severe OSA. The mean AHI was 36.27 ± 17.66/h, and the lowest oxygen saturation level (SaO_2_) was 78.6 ± 8.52%. None of the patients were diagnosed with OSAS and received continuous positive air pressure (CPAP) treatment before. As shown in Table 4, the moderate OSAS patients had highest daytime SBP (*P* < 0.05), and the severe OSAS patients had the lowest SaO_2_ (*P* < 0.05). There was no consistent significant tendency in terms of prevalence of male gender, urinary sodium, PRA, plasma and urinary aldosterone levels, and ARR with increasing severity of OSAS. The correlation analysis showed that BMI was negatively correlated with the lowest SaO_2_ (*r* = −0.318, *P* = 0.018) (Fig. 1) but not with the AHI.

**Table 4:** Clinical characteristics of patients with PA and OSAS stratified by OSA severity

	AHI 5–14 (*n* = 7)	AHI 15–29 (*n* = 21)	AHI ≥ 30 (*n* = 40)	*P* value
Age (years)	47.6 ± 14.9	46.7 ± 10.3	50.4 ± 9.2	0.39
Male sex (%)	3 (42.8%)	17 (81.0%)	29 (72.5%)	0.15
BMI (kg/m^2^)	27.5 ± 3.3	27.5 ± 2.8	27.5 ± 4.2	0.99
Antihypertensive drugs (*n*)	2 (1 to 4)	3 (3 to 4)	3 (2 to 4)	0.21
Hypertension duration (years)	6 (1 to 17)	10 (5 to 15)	10 (5 to 17.5)	0.75
Resistant hypertension (%)	2 (8.0)	9 (36.0)	14 (56.0)	0.744
24 h SBP (mm Hg)	135.2 ± 13.5	143.3 ± 14.8	134.5 ± 11.8	0.06
24 h DBP (mm Hg)	85.3 ± 10.9	92.3 ± 10.1	88.1 ± 7.7	0.13
Daytime SBP (mm Hg)	135.5 ± 14.7	146.9 ± 15.4	137.2 ± 11.6	0.03
Daytime DBP (mm Hg)	85.5 ± 12.4	94.4 ± 10.3	89.7 ± 7.1	0.06
Nighttime SBP (mm Hg)	134.8 ± 12.6	136.5 ± 14.7	129.4 ± 14.6	0.20
Nighttime DBP (mm Hg)	84.3 ± 9	87.7 ± 10.3	84.8 ± 9.7	0.55
Nocturnal systolic dip (%)	1.1 (−5.5 to −4.5)	7.2 (2.5 to −11.7)	5.4 (0.9 to −12.1)	0.12
Nocturnal diastolic dip (%)	1.4 (−5.1 to −4.9)	7.1 (3.3 to −11.4)	6.0 (1.3 to −13.0)	0.14
Serum K^+^(mmol/L)	3.4 ± 0.4	3.2 ± 0.5	3.3 ± 0.4	0.25
24 h urinary Na^+^ (µg/24 h)	190.3 ± 92.7	198.9 ± 82.8	191.8 ± 91.2	0.95
24 h urinary protein (mg/24 h)	129.3 ± 37.6	312.4 ± 416.1	138.2 ± 75.5	0.028
eGFR (mL/min·1.73 m^2^)	105.9 ± 13.4	103.9 ± 10.3	101.4 ± 7.8	0.37
LVMI (g/m^2^)	106.3 ± 20.6	109.6 ± 22.6	105.3 ± 21.8	0.80
Supine renin activity (ng/mL·h)	0.7 ± 0.8	0.6 ± 0.4	0.7 ± 0.9	0.74
Standing renin activity (ng/mL·h)	1.5 ± 1.4	1.3 ± 0.7	1.5 ± 1.5	0.89
Supine plasma aldosterone (pg/mL)	243 ± 193.1	287.5 ± 150.5	227.7 ± 117.5	0.28
Standing plasma aldosterone (pg/mL)	252.1 ± 85.4	352.6 ± 257.5	250.6 ± 132.5	0.10
Supine ARR ([pg/mL]/[ng/mL·h])	353 (294 to 1310)	497 (284 to 1134)	525 (193 to 1766)	0.69
Standing ARR ([pg/mL]/[ng/mL·h])	457 ± 684	459 ± 590	511 ± 929	0.97
24 h urinary aldosterone (μg/24 h)	18.5 ± 10.6	22.6 ± 9.9	17.6 ± 9.5	0.18
Lowest oxygen saturation (%)	82.5 ± 6.1	82.6 ± 5.6	75.3 ± 9.2	0.015

Values are indicated as means ± standard deviations or as medians (25th, 75th)

*PA* primary aldosteronism, *OSAS* obstructive sleep apnea–hypopnea syndrome, *BMI* body mass index, *SBP* systolic blood pressure, *DBP* diastolic blood pressure, *eGFR* estimated glomerular filtration rate (Modification of Diet in Renal Disease [24]), *LVMI* left ventricular mass index [25], *ARR* plasma aldosterone-to-renin activity ratio

## Discussion

The primary findings of this study were that all patients with PA with snoring were confirmed to have OSAS by PSG, with majority of them moderate-to-severe OSAS. When compared with those non-snoring patients, patients with snoring (in this study, they were actually patients with OSAS) were more commonly men, more often obese (with higher BMI values), and were also more likely to have higher diastolic blood pressure; higher levels of triglycerides, low-density lipoprotein, and serum and urinary sodium; and lower eGFR, supine plasma aldosterone levels, and ARR. When matched for age, sex, duration of hypertension, and BMI, the PA with snoring group still showed significantly reduced eGFR as well as higher serum and urinary sodium levels and lower ARR.

In recent years, the prevalence of comorbid PA and OSAS has greatly increased as screening methods have improved. The prevalence of OSAS in patients with PA has been reported as 6.7% and reached up to 59.4% in resistant hypertension patients with PA. In our study, 10% (68 cases) patients with snoring received PSG examination and all were diagnosed to have OSAS; they were symptomatic as indicated by the mean AHI superior than 30/h. Unlike the Prejbisz et al. study, all patients irrespective of their symptoms of OSAS received PSG [2], and we did not systemically do this screening in other 609 control patients with PA. It is possible that some patients with a milder form of OSAS were ignored in the initial screen and were recruited into the control group. From this point of view, the actual prevalence of OSAS in our patients with PA must be higher than the current one.

Patients with both PA and OSAS may theoretically have severe organ damage and metabolic disorder, although this has not been adequately investigated. In our study, we observed lower eGFR in snoring patients who were confirmed to have OSAS. This decrease may be related to nocturnal hypoxia as reported by the European Sleep Apnea Database cohort study that was conducted on 7770 patients and found that the lowest nocturnal SaO_2_ is an independent predictive factor of eGFR < 60 mL/min·m^2^ [15]. Recent studies found that repetitive nighttime hypoxemia and hypercapnia stimulate the secretion of aldosterone via RAAS activation [7]. Aldosterone further causes renal dysfunction by inducing glomerular sclerosis, tubulointerstitial fibrosis, and damage to glomerular mesangial cells and podocytes [16]. In addition, upper airway collapse that occurs repeatedly during sleep induces arterial hypoxia and ischemia–reperfusion injury, leading to systemic inflammatory responses, oxidative stress, and eventually renal injury [17]. Taken together, patients with PA plus OSAS may develop exacerbated renal injury. Prejbisz et al. [2] found that patients with resistant hypertension who had PA plus OSAS were more obese, had more metabolic abnormalities, and exhibited more frequent microalbuminuria and left ventricular hypertrophy than patients with either PA or OSAS alone. Among patients with resistant hypertension in our study, those complicated by OSAS were younger and more likely to be male, obese, and had higher serum and urine sodium levels; however, no difference in proteinuria or left ventricular hypertrophy was observed (data not shown).

Compared to patients with PA alone, patients with PA and snoring in our study showed milder renin suppression as characterized by a higher renin activity level and relatively lower plasma and urinary aldosterone levels and ARR. These differences were observed even after patients were matched for age, sex, duration of hypertension, and BMI. This presentation is partially associated with the activation of the RAAS pathway as a consequence of recurrent hypoxemia and hypercapnia while OSAS patients are asleep [7]. Additionally, obesity was common in patients with PA plus OSAS, as adipocytes and their derivative factors have been shown to activate the local RAAS pathway [18]. However, we did not find the AHI positively related to PRA as showed in the Di Murro’s study [3]. Patients with PA and snoring also had higher serum and urinary sodium levels. High salt intake theoretically inhibits PRA and aldosterone secretion. Besides, high salt intake increased water retention and enhanced submucosal edema and further aggravated OSAS. There exists a vicious circle. At last, the relatively lower aldosterone levels in patients with PA and snoring may be related to the pathological subtypes of PA patients. In this study, most operated PA-only patients had adenoma (51.4%), while most OSAS patients had hyperplasia (93.3%). However, the number of operated patients in the PA plus OSAS group is too small. It is generally recognized that patients with aldosterone-producing adenomas (APAs) have more florid clinical features than those with hyperplasia owing to higher aldosterone and lower serum potassium levels [19, 20]. Previous studies [21–23] reported that nearly 35–80% of APA cases contained somatic mutations, mainly in the *KCNJ5* gene. Mechanistic research has found that *KCNJ5* mutations can cause adrenal cell proliferation and promote aldosterone production. It will be interesting to further analyze the mutation status of the operated patients of both groups in a larger-scale study.

Currently, ARR is widely used in clinical practice as a screening parameter for PA, as recommended by the Endocrine Society’s clinical guidelines [10]. PA confirmation tests are usually prescribed when the ARRs are greater than their cutoff values on screening. In our study, a greater proportion of patients in the PA with snoring group showed milder suppression of renin activity and lower ARR than in the control group. It can be speculated that some patients with OSAS and unsuppressed PRA may be ignored during the PA screening that count mainly on the value of ARR; even they had higher plasma aldosterone level. To reduce the possibility of false negative screening on this group of patients, a lower ARR cutoff value might be needed. Several well-designed clinical studies are required to set an appropriate reference cutoff value for ARR in patients with OSAS. Different types of patients, including those with OSAS alone or with OSAS complicated with primary hypertension, should be included in such studies; BMI and other confounding factors must be considered as well.

In our patients with OSAS, there was as no consistent significant tendency in terms of prevalence of male gender, urinary sodium, plasma and urinary aldosterone, PRA, and ARR with increasing severity of OSAS; these results resemble that of Wolley’s study [9]. We did not find a positive correlation between PRA levels and AHI as indicated by Di Murro’s study [3]. Our analysis revealed that the BMI negatively related to the lowest oxygen saturation.

Our retrospective study included some limitations. We conducted PSG only in self-reported or witnessed snoring patients. The lack of systemic use of sleepiness questionnaires or prediction algorithms may have resulted in some patients with milder presentations of OSAS being recruited into the PA-only group. Moreover, PSG was not repeated after adrenalectomy or mineralocorticoid medical treatment. Among the 68 patients with PA plus OSAS in our study, only 12 used CPAP treatment after diagnosis, and 6 of them persist on treatment 1 year later. Most patients felt uncomfortable wearing the mask during try-out and discourage them to continue the treatment.

In conclusion, patients with PA and snoring had higher BMI, diastolic blood pressure, serum and urinary sodium, and renin activity, as well as lower eGFR, aldosterone levels, and ARR than patients with PA alone. The majority of patients with snoring were confirmed to have moderate-to-severe OSAS. It is essential to set a reference ARR range for patients with snoring or OSAS when screening for PA. Additionally, the prognosis of patients with PA plus OSAS receiving CPAP treatment, spironolactone, or adrenalectomy requires further investigation.

### Summary Table

#### What is known about the topic?

- OSAS induces the activation of the sympathetic nervous system and the renin–angiotensin–aldosterone system (RAAS). Currently, the aldosterone–to–renin ratio (ARR) is widely used in clinical practice as a screening parameter for PA.
- Both PA and OSAS can result in serious cardiovascular and renal diseases, as well as metabolic syndrome.

#### What this study adds

- Patients with PA and snoring in our study showed milder renin suppression as characterized by a higher renin activity level and relatively lower plasma and urinary aldosterone levels and ARR.
- Patients with PA and snoring who confirmed to have OSAS by PSG have severe organ damage and metabolic disorder with higher BMI, diastolic blood pressure, and lower eGFR.

## References

[1] Born-Frontsberg E, Reincke M, Rump LC, Hahner S, Diederich S, Lorenz R (2009). Cardiovascular and cerebrovascular comorbidities of hypokalemic and normokalemic primary aldosteronism: results of the German Conn’s Registry. J Clin Endocrinol Metab.

[2] Prejbisz A, Florczak E, Klisiewicz A, Dobrowolski P, Janaszek-Sitkowska H, Bieleń P (2013). Relationship between primary aldosteronism and obstructive sleep apnoea, metabolic abnormalities and cardiac structure in patients with resistant hypertension. Endokrynol Pol.

[3] Di Murro A, Petramala L, Cotesta D, Zinnamosca L, Crescenzi E, Marinelli C (2010). Renin-angiotensin-aldosterone system in patients with sleep apnoea: prevalence of primary aldosteronism. J Renin Angiotensin Aldosterone Syst.

[4] Prejbisz A, Kołodziejczyk-Kruk S, Lenders JWM, Januszewicz A (2017). Primary aldosteronism and obstructive sleep apnea: is this a bidirectional relationship?. Horm Metab Res.

[5] Gonzaga CC, Gaddam KK, Ahmed MI, Pimenta E, Thomas SJ, Harding SM (2010). Severity of obstructive sleep apnea is related to aldosterone status in subjects with resistant hypertension. J Clin Sleep Med.

[6] White LH, Bradley TD, Logan AG (2015). Pathogenesis of obstructive sleep apnoea in hypertensive patients: role of fluid retention and nocturnal rostral fluid shift. J Hum Hypertens.

[7] Jin ZN, Wei YX (2016). Meta-analysis of effects of obstructive sleep apnea on the renin-angiotensin aldosterone system. J Geriatr Cardiol.

[8] Funder JW, Carey RM, Mantero F, Murad MH, Reincke M, Shibata H (2016). The management of primary aldosteronism: case detection, diagnosis, and treatment: an Endocrine Society clinical practice guideline. J Clin Endocrinol Metab.

[9] Wolley MJ, Pimenta E, Calhoun D, Gordon RD, Cowley D (2017). Treatment of primary aldosteronism is associated with a reduction in the severity of obstructive sleep apnea. J Hum Hypertens.

[10] Funder JW, Carey RM, Fardella C, Gomez-Sanchez CE, Mantero F, Stowasser M (2008). Case detection, diagnosis, and treatment of patients with primary aldosteronism: An Endocrine Society clinical practice guideline. J Clin Endocrinol Metab.

[11] Zhang Y, Niu W, Zheng F, Zhang H, Zhou W, Shen Z (2017). Identifying unilateral disease in Chinese patients with primary aldosteronism by using a modified prediction score. J Hypertens.

[12] Chen SX, Du YL, Zhang J, Gong YC, Hu YR, Chu SL (2006). Aldosterone-to-renin ratio threshold for screening primary aldosteronism in Chinese hypertensive patients. Chin J Cardiovasc Dis.

[13] Rossi GP, Auchus RJ, Brown M, Lenders JWM, Naruse M, Plouin PF (2014). An expert consensus statement on use of adrenal vein sampling for the subtyping of primary aldosteronism. Hypertension.

[14] Kushida CA, Morgenthaler TI, Littner MR, Alessi CA, Bailey D, Coleman J (2006). Practice parameters for the treatment of snoring and obstructive sleep apnea with oral appliances: an update for 2005. Sleep.

[15] Marrone O, Battaglia S, Steiropoulos P, Basoglu OK, Kvamme JA, Ryan S (2016). Chronic kidney disease in European patients with obstructive sleep apnea: the ESADA cohort study. J Sleep Res.

[16] Rafiq K, Hitomi H, Nakano D, Nishiyama A (2011). Pathophysiological roles of aldosterone and mineralocorticoid receptor in the kidney. J Pharm Sci.

[17] Abuyassin B, Sharma K, Ayas NT, Laher I (2015). Obstructive sleep apnea and kidney disease: a potential bidirectional relationship?. J Clin Sleep Med.

[18] Dinh Cat AN, Friederich-Persson M, White A, Touyz RM (2016). Adipocytes, aldosterone and obesity-related hypertension. J Mol Endocrinol.

[19] Somlóová Z, Indra T, Rosa J, Petrák O, Strauch B (2012). Have main types of primary aldosteronism different phenotype?. Physiol Res.

[20] Wu JC, Tang ZY, Zhang W, Ling DY, Hou RF, Wang WQ (2006). Clinical characteristics and surgery outcomes of unilateral nodular adrenal hyperplasia in primary aldosteronism: study of 145 cases. Zhonghua Yi Xue Za Zhi.

[21] Dutta RK, Söderkvist P, Gimm O (2016). Genetics of primary hyperaldosteronism. Endocr Relat Cancer.

[22] Zheng FF, Zhu LM, Nie AF, Li XY, Lin JR, Zhang K (2015). Clinical characteristics of somatic mutations in Chinese patients with aldosterone-producing adenoma. Hypertension.

[23] Aragao-Santiago L, Gomez-Sanchez CE, Mulatero P, Spyroglou A, Reincke M, Williams TA (2017). Mouse models of primary aldosteronism: from physiology to pathophysiology. Endocrinology.

[24] National Kidney Foundation. (2002). K/DOQI clinical practice guidelines for chronic kidney disease: evaluation, classification, and stratification. Am J Kidney Dis.

[25] Devereux RB, Alonso DR, Lutas EM, Gottlieb GJ, Campo E, Sachs I (1986). Echocardiographic assessment of left ventricular hypertrophy: comparison to necropsy findings. Am J Cardiol.

